# Fingerprinting-Based Indoor Localization Using Interpolated Preprocessed CSI Phases and Bayesian Tracking

**DOI:** 10.3390/s20102854

**Published:** 2020-05-18

**Authors:** Wenxu Wang, Damián Marelli, Minyue Fu

**Affiliations:** 1 School of Automation, Guangdong University of Technology, Guangzhou 510006, China; wangwenxu0909@126.com (W.W.); minyue.fu@newcastle.edu.au (M.F.); 2 French Argentine International Center for Information and Systems Sciences, National Scientific and Technical Research Council, 2000 Rosario, Argentina; 3 School of Electrical Engineering and Computer Science, University of Newcastle, Callaghan NSW 2308, Australia

**Keywords:** indoor localization, CSI, fingerprinting, Bayesian tracking

## Abstract

Indoor positioning using Wi-Fi signals is an economic technique. Its drawback is that multipath propagation distorts these signals, leading to an inaccurate localization. An approach to improve the positioning accuracy consists of using fingerprints based on channel state information (CSI). Following this line, we propose a new positioning method which consists of three stages. In the first stage, which is run during initialization, we build a model for the fingerprints of the environment in which we do localization. This model permits obtaining a precise interpolation of fingerprints at positions where a fingerprint measurement is not available. In the second stage, we use this model to obtain a preliminary position estimate based only on the fingerprint measured at the receiver’s location. Finally, in the third stage, we combine this preliminary estimation with the dynamical model of the receiver’s motion to obtain the final estimation. We compare the localization accuracy of the proposed method with other rival methods in two scenarios, namely, when fingerprints used for localization are similar to those used for initialization, and when they differ due to alterations in the environment. Our experiments show that the proposed method outperforms its rivals in both scenarios.

## 1. Introduction

The global positioning system (GPS) permits solving the positioning problem in a reliable manner. However, this approach is limited to outdoor environments, since GPS signals do not reach indoor receivers. A number of techniques are available for indoor positioning systems [1,2]. These techniques include the use of ultra-wideband signals [3,4,5], Wi-Fi signals [6,7,8], bluetooth signals [9,10], radio-frequency identification [11,12,13], odometry measurements [14], etc. However, the accuracy of positioning systems based on these techniques is severely affected by the multipath propagation of signals. Therefore, obtaining a high-precision and reliable indoor positioning method has become the main research problem in various location-based services.

A popular technique consists of using Wi-Fi signals for indoor positioning. The main reason for doing so is the widespread and low cost of these signals in comparison with other alternatives, e.g., Zigbee, Bluetooth, ultrasound, ultra-wideband, etc. There are two main approaches for indoor positioning using Wi-Fi signals, namely, geometric mapping and fingerprinting [6]. Geometric mapping is based on the estimation of geometric parameters, such as distance or angles with respect to certain reference points [15,16]. The problem with this approach is, as we mentioned earlier, the irregularities in the signals caused by multipath effects. To go around this, the fingerprint approach builds a database of certain features obtained from the received Wi-Fi signals, depicting a unique pattern based on its location. The positioning problem then becomes one of finding the best match for the signal received at an unknown location from the information available within the database.

For an indoor positioning system, which kind of signal feature is selected as a fingerprint is critical, as it must faithfully represent its location. A popular choice for this feature is a vector of received signal strength indicators (RSSIs) from multiple Wi-Fi access points (APs) [17,18]. Then, the position is estimated as the one from the database with the highest similarity score with the fingerprint of the received signal. The disadvantage of this method is that each location is only matched to one within the database. To solve this, Youssef [19] estimates the position by building its conditional probability given the available measurements. A theoretical backing for this approach is given by the fact that RSSI measurements can be approximately considered normally distributed with negligible accuracy loss [20].

The advantages of using RSSI as fingerprints are its simplicity and low computational requirements. However, since the database only stores signal features at a finite number of points, the accuracy drops significantly at other points. This is because RSSI does not contain all the information available in the Wi-Fi signal, which could be used to improve the positioning accuracy. The whole available information appears in the channel state information (CSI). This is a complex vector whose entries represent the gain (amplitude and phase) of each subcarrier of the OFDM channel [6]. In [21], CSI was used to propose the fine-grained indoor localization (FILA) method. In this method, the fingerprint is chosen to be the square sum of CSI amplitudes. A popular choice of fingerprint consists of using the real vector formed by the amplitudes of each subcarrier [22,23,24]. However, the instability of CSI amplitudes limit the accuracy achievable with this kind of fingerprints. In an attempt to go around this, inter-subcarrier phase differences are used as fingerprints in [25]. A drawback of this approach is that CSI phases are highly sensitive to hardware imperfections. To cope with this, a phase calibration method was proposed in [26] to compensate for hardware effects.

Regardless of the choice of fingerprint, two approaches are available for estimating the position. The first one consists in dividing the area into regions, and using a classifier, based on machine learning techniques, to decide the region at which the receiver is located. In [27], this is done using a neural network, and in [28], a random forest model. A drawback of these methods is that the positioning accuracy is determined by the regions’ size.

The second positioning approach aims at solving the above limitation by interpolating a position from those available within the fingerprint database. In [21], this is done using Pearson correlation coefficients between the measured fingerprints at the unknown location and the fingerprints from the database. In [26], this is done using a three-hidden-layer deep network, a deep autoencoder is used in [29,30], a deep convolutional neural networks in [31] and a k-nearest neighbor average in [25]. Nevertheless, a common problem with these methods is that their inaccuracy is still significant at locations outside the database.

For moving targets, the above positioning techniques are used for carrying out a Bayesian tracking task, which uses, at each sample time, all available measurements to provide a position estimate [32,33]. The case in which positioning is based on Wi-Fi signals, the information provided by these signals are often combined (fused) with certain extra information, such as infra-red motion sensors [34], inertial sensors [35], orientation sensors and landmarks [36].

In this work, a new fingerprint method for indoor positioning and Bayesian tracking is proposed, based on CSI. Following [26], we also calibrate phases. However, instead of using them as fingerprints, we use their differences with respect to the first subcarrier. We do so because these phase differences change more smoothly than absolute phases as we move along a straight line. This largely simplifies the task of interpolating fingerprints at locations outside the database. To do the interpolation, we construct a model of the room’s fingerprints, i.e., a function mapping positions within the room to fingerprints. We do so using a Gaussian kernel expansion. Using this model, the positioning task is split into two steps. In the first one, we obtain a preliminary estimate of the position using only the fingerprint measured by the receiver. To this end, we use the maximum likelihood criterion. In the second stage, we combine this preliminary estimation with a dynamic model of the receiver’s motion, to obtain the final estimation. We do so using a Bayesian tracking technique. The most popular among these techniques are extended Kalman filtering and particle filtering. The advantage of the former is its computational efficiency. However, since it is based on linearizing a non-linear model, it is often inaccurate to the extent that it can lead to the instability of the estimate. Particle filtering, on the other hand, can be made arbitrarily accurate. However, its computational complexity makes it often unfeasible for real-time applications. In order to obtain a reliable and numerically efficient Bayesian tracking implementation, we use a recently proposed technique called maximum likelihood Kalman filtering [37]. It uses the maximum likelihood estimate obtained in the first step, guarantees the stability of the estimate and is asymptotically optimal as the dimension of the fingerprint vector tends to infinity [37]. To summarize, the contribution of our paper is to effectively combine the use of phase information, its calibration and phase differences for fingerprints, and to apply Gaussian kernel modeling and maximum likelihood Kalman filtering for positioning. The resulting algorithm is shown, via experiments, to have more accurate position estimates, in comparison with other available algorithms, at locations outside the database.

The rest of this paper is organized as follows. In Section 2 we describe how we build fingerprints. In Section 3 we formulate the positioning problem. The proposed localization method is introduced in Section 4. Experimental results are given in Section 5 and concluding remarks in Section 6.

## 2. Choice of Fingerprints

### 2.1. Channel State Information

In an OFDM system, the signal received from a multipath channel can be described as

(1) r=Cs+n,

where $r=\left[r_{1},\cdots ,r_{L}\right]\in C^{L}$ and $s=\left[s_{1},\cdots ,s_{L}\right]\in C^{L}$ represent the received and transmitted signal vectors, respectively, *L* is the number of subcarriers, *n* is additive noise, $C=diag\left(c\right)\in C^{L\times L}$ is the channel frequency response (CFR) matrix, with $c=\left[c_{1},\cdots ,c_{L}\right]$ and $c_{m}$ denoting the channel’s gain at the *m*-th subcarrier.

We call vector *c* the CSI. According to the 802.11n protocol, this vector is used to recover *s* from *r* by equalizing the channel distortion caused by multipaths. Hence, the CSI *c* is readily available in every OFDM receiver.

### 2.2. Phase Information

The CSI value $c_{m}$, at each subcarrier m=1,⋯,L, is a complex number. Most CSI-based fingerprint methods choose amplitudes $\left|c_{m}\right|$ as their fingerprints. However, Sen et al. [38] proposed a linear calibration method for the (raw) phase information $∠c_{m}$. As we show below, the calibrated phases so obtained are more stable than amplitudes, in the sense that they incur smaller changes between consecutive measurements. We summarize this calibration method below.

We assume that raw CSI measurements *c* have certain packet-by-packet random phase shifts due to synchronization errors at the receiver. More precisely, let $\theta_{m}=∠c_{m}$ denote the raw phase of the *m*-th subcarrier and $\phi_{m}$ denote the ideal phase that we would have received if there were no random phase shifts. We then have

(2) $$\theta_{m}=\phi_{m}+\frac{2\pi }{L\Delta t}l_{m}+q,$$

where *q* denotes the unknown center frequency shift and Δt denotes the delay due to both, packet detection delay (PDD) and sampling frequency offset (SFO). By $l_{m}$, we denote the *m*-th entry of the subcarrier index vector *l*, and, as above, *L* is the number of subcarriers. For example, in the 20 MHz bandwidth 802.11n protocol, L=56 and

$$l={\left[-28,-27,\ldots ,-2,-1,1,2,\ldots ,27,28\right]}^{⊤}.$$

In order to compensate for the effects of random phase shifts, we use

(3) $$\phi_{m}=\theta_{m}-ol_{m}-q,$$

with

$$\begin{array}{cc} o & =\frac{\theta_{L}-\theta_{1}}{l_{L}-l_{1}},\\ q & =\frac{1}{L}\sum_{m=1}^{L}\theta_{m}. \end{array}$$

**Figure 1:**
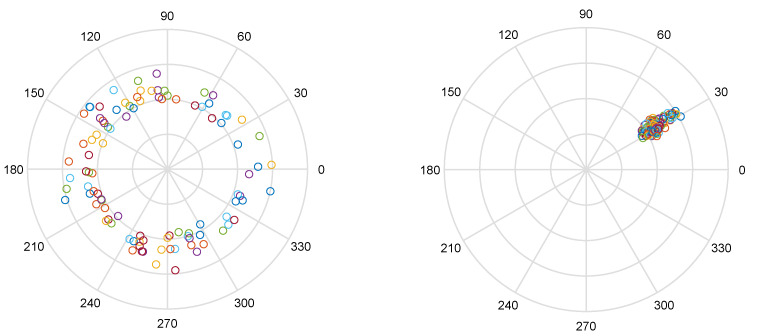
(**Left**) Complex values, in polar coordinates, corresponding to subcarrier m=1 of 100 raw channel state information (CSI) measurements. (**Right**) The same values after phase calibration.

Figure 1 illustrates the effect of phase calibration. On the left, we see 100 raw measurements of the CSI at subcarrier m=1, and on the right, the same CSI measurements with calibrated phases. We see how, after calibration, phases are more stable than amplitudes.

### 2.3. Phase Differences

**Figure 2:**
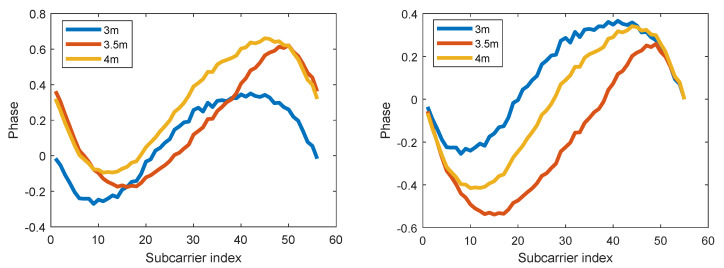
(**Left**) Compensated CSI phase at three adjacent positions. (**Right**) Phase differences at the same positions.

The calibrated phase is stable at a fixed position. However, it changes drastically from one position to another. This occurs because, theoretically, for a subcarrier at 2.4 GHz, the phase goes through an entire cycle in a wavelength distance (about 12.5 cm). This is much shorter than the distance between positions used to build the fingerprint database. To go around this, instead of using the compensated phases $\phi_{m}$, m=1,⋯,L, we use their differences $\psi_{m}=\phi_{m+1}-\phi_{1}$, m=1,⋯,L−1, with respect to the first component, and unwrap them so as to avoid that jumps $\left|\psi_{m+1}-\psi_{m}\right|$, m=1,⋯,L−2, are bigger than or equal to π. This is equivalent to consider that the frequency of the first subcarrier is zero, and the frequencies of each other subcarriers are 312.5 KHz, 625 KHz,..., 20 MHz. Then, the smallest wavelength among these subcarriers becomes $\left(300\times 10^{6}\;m/s\right)/\left(20\times 10^{6}\;\mathrm{Hz}\right)=15\;m$. Figure 2, shows how phase differences change more consistently than plain phases in a sequence of three points, arranged in a straight line, 0.5 m away from each other.

We use $y_{m}$, m=1,⋯,L−1, to denote the resulting unwrapped calibrated phase differences (UCPDs), and use them as fingerprints for indoor localization.

## 3. Problem Description

We consider a room ($R^{2}$) having *A* Wi-Fi access points (APs) with *B* antennas. At time t∈N, a receiver, having *D* antennas, is located at $x\left(t\right)\in R^{2}$, within the room. The OFDM channel establishing the communication between each AP and each receiver’s antenna, has *L*, subcarriers, whose frequencies are $f_{m}\in R$, m=1,⋯,L. The UCPD of the *m*-th subcarrier of the channel from AP *a* antenna *b* to receiver antenna *d* is denoted by $y_{m}^{\left(a,b,d\right)}\left(t\right)$, m=1,⋯,L−1. Let $y^{\left(a,b,d\right)}\left(t\right)=\left[y_{1}^{\left(a,b,d\right)}\left(t\right),\cdots y_{L-1}^{\left(a,b,d\right)}\left(t\right)\right]\in R^{L-1}$ be the set of all UCPDs of the same channel, $y^{(a,b)⊤}\left(t\right)=\left[y^{(a,b,1)⊤}\left(t\right),\cdots ,y^{(a,b,D)⊤}\left(t\right)\right]$ be the set of all UCPDs from AP *a* antenna *b* and $y^{⊤}\left(t\right)=\left[y^{(1,1)⊤}\left(t\right),\cdots ,y^{(A,1)⊤}\left(t\right),\ldots ,y^{(1,B)⊤}\left(t\right),\cdots ,y^{(A,B)⊤}\left(t\right)\right]$ be the set of all UCPDs. The fingerprint at x(t) is given by $y\left(t\right)\in R^{N}$, with N=(L−1)ABD.

We assume that x(t) and y(t) are generated by the following model:

(4) $$\begin{array}{cc} \xi (t+1) & =F\xi (t)+w(t), \end{array}$$

(5) $$\begin{array}{cc} y(t)= & h\left(J\xi (t)\right)+\iota \left(t\right), \end{array}$$

where the state $\xi \left(t\right)={\left[x^{⊤}\left(t\right),{\dot{x}}^{⊤}\left(t\right)\right]}^{⊤}$ contains the position and velocity of the receiver at time *t*, $F\in R^{4\times 4}$ is the state-transition matrix, w(t)∼N(0,Q) is the process noise, ι(t)∼N(0,R) is the measurement noise, w(t) and ι(t) are white and jointly independent, and $J={\left[I,0\right]}^{⊤}$, with I being the identity matrix, is a selection matrix such that Jξ(t)=x(t).

We assume that matrices *F* and *Q*, as well as the fingerprints y(t) are known. On the other hand, matrix *R* and the map $\eta :R^{2}\to R^{N}$ are not known.

**Problem** **1.** *Based on the above model and assumptions, our goal is to estimate, at each t∈N, the receiver’s location x(t).*

## 4. Proposed Positioning Method

The proposed method is formed by three stages. The first one is called initialization. In this stage, we estimate the fingerprint model $\eta :R^{2}\to R^{N}$. The second one (static positioning), uses the estimated model and the output Equation (5) to obtain a first estimate $\overset{ˇ}{x}\left(t\right)$ of the receiver’s location x(t), at time t∈N, given the fingerprint y(t). In the third stage (dynamic positioning), the estimated location $\overset{ˇ}{x}\left(t\right)$ obtained in the second stage is combined with the dynamic motion model (4) to obtain the final estimation $\hat{x}\left(t\right)$ of x(t).

### 4.1. Fingerprint Modeling

The goal of this stage is to obtain estimates of both, the map $h:R^{2}\to R^{N}$ and the matrix $R\in R^{N\times N}$. To do so, we rely on a collection of points $p_{i}\in R^{2}$, i=1,⋯,I, at each of which *K* measurements $g_{i,k}\in R^{N}$, k=1,⋯,K of the fingerprint are available. The ground truth of each $p_{i}$ is known.

It follows from (5) that

(6) $$g_{i,k}=h\left(p_{i}\right)+\iota_{i,k},$$

with $\iota_{i,k}∼N\left(0,Q\right)$ being jointly independent. Let ${\bar{g}}_{i}=\frac{1}{K}\sum_{k=1}^{K}g_{i,k}$. We then have

(7) $$\begin{array}{cc} {\bar{g}}_{i} & =h\left(p_{i}\right)+{\bar{\iota }}_{i},\\ {\bar{\iota }}_{i} & =\frac{1}{K}\sum_{k=1}^{K}\iota_{i,k}. \end{array}$$

Since ${\bar{\iota }}_{i}∼N\left(0,\frac{1}{K}Q\right)$, for large *K* we can do the following approximation

(8) $${\bar{g}}_{i}\approx h\left(p_{i}\right).$$

We can then obtain an estimate $\hat{\eta }$ of η by approxmating h(p) using a Gaussian kernel expansion of the form

(9) $$h\left(p\right)\approx \hat{h}\left(p,\alpha \right)=\sum_{i=1}^{I}\alpha_{i}\exp\left(-\gamma {∥p-p_{i}∥}^{2}\right),$$

where $\alpha ={\left[\alpha_{1},\cdots ,\alpha_{I}\right]}^{⊤}$. Following [39], we choose $\gamma =\frac{1}{2}I^{1/3}$. Then, using (8), we estimate α as follows

(10) $$\hat{\alpha }=\underset{\alpha }{\arg\min}\sum_{i=1}^{I}{\left[\hat{h}\left(p_{i},\alpha \right)-{\bar{g}}_{i}\right]}^{2}.$$

Finally, in order to obtain an estimate $\hat{R}$ of *R*, we do

(11) $$\hat{R}\approx \frac{1}{I}\sum_{i=1}^{I}\frac{1}{K-1}\sum_{k=1}^{K}\left(g_{i,k}-{\bar{g}}_{i}\right){\left(g_{i,k}-{\bar{g}}_{i}\right)}^{⊤}.$$

### 4.2. Static Positioning

Knowing the estimate $\hat{\alpha }$, we can replace (5) by

(12) $$y\left(t\right)=\hat{h}\left(J\xi \left(t\right),\hat{\alpha }\right)+\hat{\iota }\left(t\right).$$

with $\hat{\iota }\left(t\right)∼N\left(0,\hat{R}\right)$. We can then obtain an estimate $\overset{ˇ}{x}\left(t\right)$ of x(t) using the maximum likelihood criterion, i.e.,

(13) $$\overset{ˇ}{x}\left(t\right)=\underset{x}{\arg\max}L_{t}\left(x\right),$$

where

(14) $$L_{t}\left(x\right)=\log p\left(y(t)|J\xi (t)=x\right),$$

denotes the log-likelihood function and

(15) $$p\left(y(t)|J\xi (t)=x\right)=N\left(y\left(t\right);\hat{h}\left(x,\hat{\alpha }\right),\hat{R}\right).$$

### 4.3. Dynamic Positioning

We aim to use a Bayesian tracking method to estimate ξ(t) based on the model (4), (12), and then obtaining x(t) from the estimated value of ξ(t). In principle, this could be done using the typical approaches for Bayesian tracking, namely, extended Kalman filtering (EKF) and particle filtering (PF). However, since EKF is based on linearizing the non-linear measurement Equation (5), its result is inaccurate and it often causes instability of the estimate. On the other hand, while this problem can be solved using PF, which can be made arbitrarily accurate by using enough particles, the number of required particles makes this method impractical for real-time applications. In order to obtain an accurate and numerically tractable Bayesian tracking implementation, we use a method called maximum likelihood Kalman filter [37]. We summarize it below.

We want to compute

(16) $$\begin{array}{cc} \hat{\xi }\left(t\right) & =E\left\{\xi (t)|Y(t)\right\}\\ \; & =\int \xi \left(t\right)p\left(\xi (t)|Y(t)\right)d\xi \left(t\right), \end{array}$$

where $Y\left(t\right)=\left\{y(1),\cdots ,y(t)\right\}$. In order to compute $p\left(\xi (t)|Y(t)\right)$ we alternate two steps, namely, prediction and update, which are described below.

#### 4.3.1. Prediction

In this step we assume that $\xi \left(t\right)|Y\left(t\right)∼N\left(\mu (t|t),\Sigma (t|t)\right)$, with μ(t|t) and Σ(t|t) known. We aim to compute μ(t+1|t) and Σ(t+1|t) such that $\xi \left(t+1|t\right)|Y\left(t\right)∼N\left(\mu (t+1|t),\Sigma (t+1|t)\right)$. We do so using a typical Kalman filtering prediction step, i.e.,

(17) $$\begin{array}{cc} \mu (t+1|t) & =F\mu (t|t),\\ \Sigma (t+1|t) & =F\Sigma \left(t|t\right)F^{⊤}+Q. \end{array}$$

#### 4.3.2. Update

In this case, we assume that $\xi \left(t\right)|Y\left(t-1\right)∼N\left(\mu (t|t-1),\Sigma (t|t-1)\right)$, with μ(t|t−1) and Σ(t|t−1) known. We aim to compute μ(t|t) and Σ(t|t) such that $\xi \left(t\right)|Y\left(t\right)∼N\left(\mu (t|t),\Sigma (t|t)\right)$. In this case, we cannot do a typical Kalman filtering step, because the measurement equation is nonlinear. To go around this, we use the estimate $\overset{ˇ}{x}\left(t\right)$ obtained by the static positioning method described in Section 4.2. More precisely, we compute

(18) $$\begin{array}{cc} \mu (t|t) & =\Sigma \left(t|t\right)\left(\Sigma^{-1}\left(t|t-1\right)\mu \left(t|t-1\right)+\Lambda \left(t\right)\lambda \left(t\right)\right),\\ \Sigma (t|t) & ={\left(\Sigma^{-1}\left(t|t-1\right)+\Lambda \left(t\right)\right)}^{-1}, \end{array}$$

where

$$\begin{array}{cc} \lambda (t) & =J^{⊤}\overset{ˇ}{x}\left(t\right),\\ \Lambda (t) & =J^{⊤}\overset{ˇ}{C}\left(t\right)J, \end{array}$$

and

$$\overset{ˇ}{C}\left(t\right)=-\nabla^{2}L_{t}\left(\overset{ˇ}{x}\left(t\right)\right),$$

with $\nabla^{2}L_{t}\left(x\right)$ denoting the Hessian of $L_{t}$ evaluated at *x* (recall that $\overset{ˇ}{x}\left(t\right)$ is computed from (13)).

#### 4.3.3. Final Step

Using the above, at each *t*, we can compute μ(t|t) and Σ(t|t) such that $\xi \left(t\right)|Y\left(t\right)∼N\left(\mu (t|t),\Sigma (t|t)\right)$. Then, using (16), we obtain

(19) $$\hat{\xi }\left(t\right)=\mu \left(t|t\right),$$

and finally

(20) $$\hat{x}\left(t\right)=J\mu \left(t|t\right).$$

## 5. Experiments

### 5.1. Static Positioning

In this section, we will experimentally compare our proposed method with three rival methods, namely, the FILA method [21], the DeepFi method [22] (amplitude-based fingerprinting), and the PhaseFi method [26] (Phase-based fingerprinting), described in Section 1. We use TP-Link WDR4310 routers, having the OpenWrt platform installed, and working at a package rate of 100 packets per second. We obtain CSI measurements from the Atheros CSI Tool [40]. For every AP/receiving antenna pair, this tool produces a complex vector whose entries are the amplitude and phase of certain subcarriers from the IEEE 802.11n standard. More precisely, for a 20 MHz channel, values are available for 56 subcarriers; for a 40 MHz channel, values are available for 114 subcarriers. 10 bits are used to represent both, the real and imaginary parts of each subcarrier’s gain. The ground truth position of the receiver is obtained using a motion capture camera, which we believe to be sufficiently reliable for our testing purposes.

**Figure 3:**
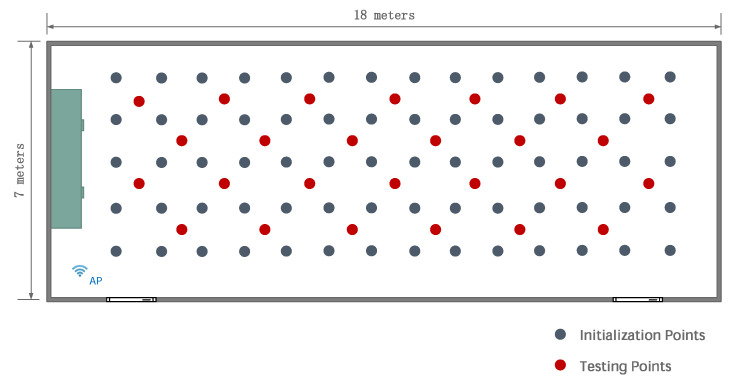
Empty room layout for method comparison. The wireless access point is placed in the lower-left corner.

**Figure 4:**
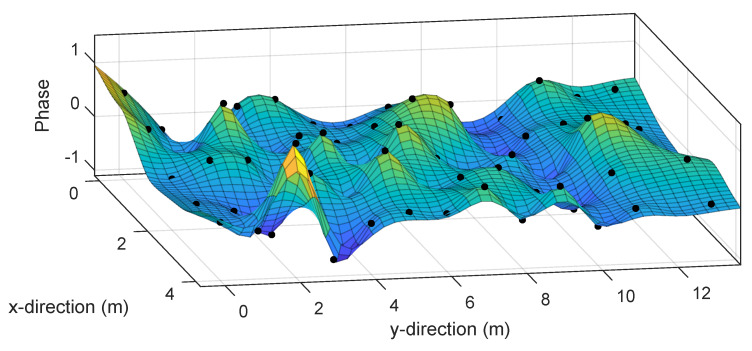
First entry of the averaged fingerprint ${\bar{g}}_{i}$ (dots) and the value yield by the model $\hat{h}\left(p,\hat{\alpha }\right)$.

We use two experimental setups. The first one is an empty room. This setup permits evaluating the positioning performance of the different methods in the ideal situation in which fingerprints used for positioning are as similar as possible to those used during the initialization stage. The setup is depicted in Figure 3, showing the wireless access point in a corner. The figure also shows the I=70 positions (marked as initialization points) used as ground truth positions for constructing the fingerprint model. In addition, there are 26 points for testing the static and dynamic positioning algorithms. Figure 4 shows the first entry of the averaged fingerprints ${\bar{g}}_{i}$ at each initialization point $p_{i}$, i=1,⋯,I, together with the value yield by the estimated model $\hat{h}\left(p,\hat{\alpha }\right)$.

**Figure 5:**
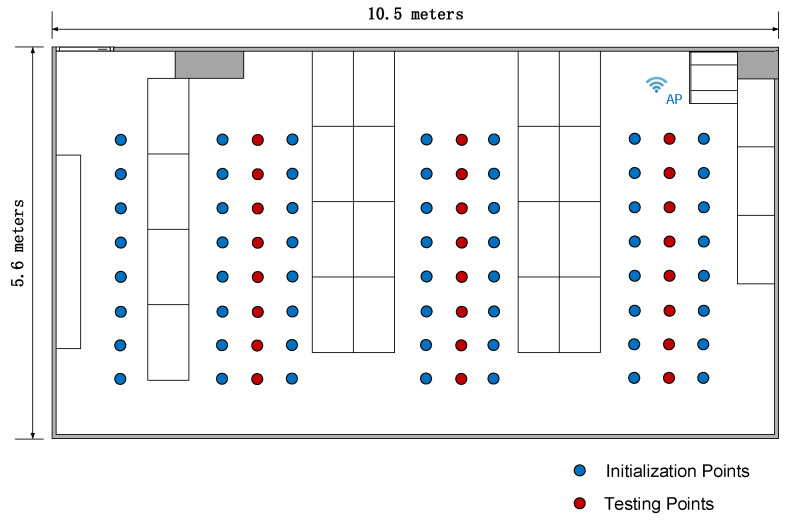
Office layout for method comparison. The wireless access point is placed in the upper-right corner, and rectangles represent desks.

The second setup is an office including people as well as objects like desks, chairs and lab benches. This setup permits evaluating the robustness of the different methods, in the sense of how the performance deteriorates when fingerprints differ from those used to build the model during initialization. The setup is depicted in Figure 5, showing desks represented by rectangles, the wireless access point, the positions of the I=56 initialization points and 24 testing points.

**Figure 6:**
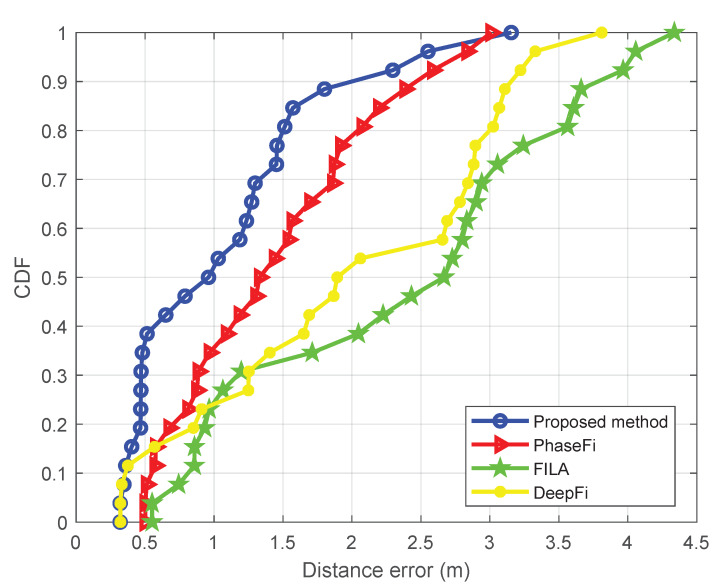
Positioning error CDF (empty room).

**Figure 7:**
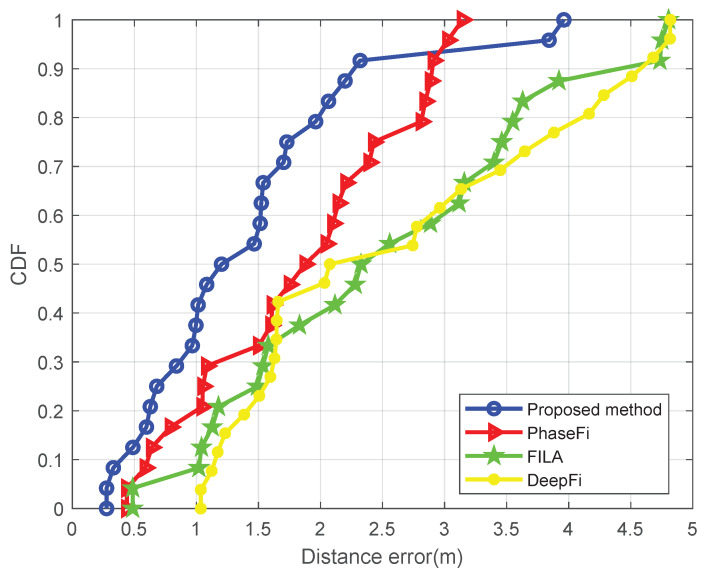
Positioning error CDF (office).

Figure 6 and Figure 7 show the cumulative distribution function (CDF) of the positioning error (in meters), (i.e., the relative number of points at which the localization error is smaller than each distance error in the horizontal axis) for the aforementioned four methods and the two setups. Table 1 and Table 2 show the minimum and mean positioning errors also for the four methods and both setups. These results show that our proposed method significantly outperforms the FILA, DeepFi and PhaseFi methods in both scenarios.

### 5.2. Dynamic Positioning

The dynamic positioning method makes use of the state-transition Equation (4). In order to design this state equation, we use the following model

(21) $$\begin{array}{cc} p_{x}\left(t+1\right) & =p_{x}\left(t\right)+Tv_{x}\left(t\right)+w_{x}\left(t\right),\\ p_{y}\left(t+1\right) & =p_{y}\left(t\right)+Tv_{y}\left(t\right)+w_{y}\left(t\right),\\ v_{x}\left(t+1\right) & =v_{x}\left(t\right)+\iota_{x}\left(t\right),\\ v_{y}\left(t+1\right) & =v_{y}\left(t\right)+\iota_{y}\left(t\right), \end{array}$$

where $p_{\zeta }$ and $v_{\zeta }$, ζ∈{x,y}, denote the position and velocity, respectively, in the ζ axis. We choose T=1 (seconds). We assume that $w_{x}$, $w_{y}$, $\iota_{x}$ and $\iota_{y}$ are mutually independent, $w_{x}∼w_{y}∼N\left(0,\sigma_{p}^{2}\right)$, with $\sigma_{p}^{2}=10^{-4}$, and $\iota_{x}∼\iota_{y}∼N\left(0,\sigma_{\iota }^{2}\right)$, with $\sigma_{\iota }^{2}=2.5\times 10^{-4}$. We therefore have

$$F=\left[\begin{array}{cccc} 1 & 0 & T & 0\\ 0 & 1 & 0 & T\\ 0 & 0 & 1 & 0\\ 0 & 0 & 0 & 1 \end{array}\right],\;\mathrm{and}\;Q=\mathrm{diag}\left(\sigma_{p}^{2},\sigma_{p}^{2},\sigma_{\iota }^{2},\sigma_{\iota }^{2}\right).$$

**Figure 8:**
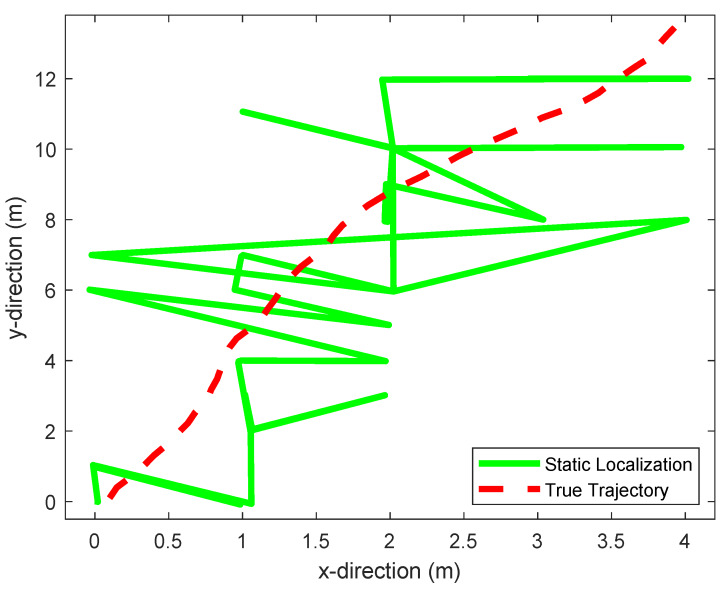
Static positioning result.

**Figure 9:**
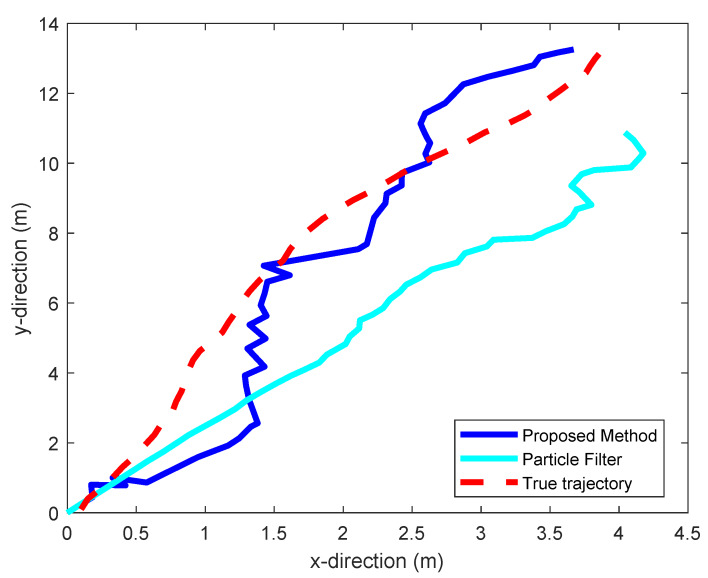
Comparison of the dynamic positioning results yield by particle filtering and our proposed dynamic positioning method.

To evaluate this performance, we moved the receiver over a trajectory that is well modeled by this equation. This trajectory is shown in red in Figure 8 and Figure 9. Figure 8 shows the trajectory estimated by our static positioning method. Figure 9 shows the one estimated using a particle filter [41], and the one estimated by our proposed dynamic positioning method based on the maximum likelihood Kalman filter. Table 3 shows the maximum tracking errors, as well as their means, given by the three methods. We see that the proposed dynamic positioning method offers a significant accuracy improvement over its static counterpart, as well as over particle filtering.

## 6. Conclusions

We proposed a new fingerprint-based method for indoor localization based on CSI. The proposed method uses unwrapped calibrated phase differences as fingerprints, and consists of three stages. In the first one (fingerprint modeling), a model of the room’s fingerprints is constructed. In the second stage (static positioning) we use this model, together with the maximum likelihood criterion, to obtain a preliminary position estimate based only on the fingerprint measured at the receiver. Finally, in the third stage (dynamic positioning), we use a maximum likelihood Kalman filter to combine this preliminary estimate with a dynamic model for the receiver’s motion, to obtain the final estimate. We present experimental results comparing the localization accuracy of the proposed method with that of other rival methods. This is done in two scenarios, namely, in the ideal one in which fingerprints used for localization are similar to those used for initialization, and in a more realistic scenario, in which these fingerprints differ due to alterations in the environment. Our experiments show that our method is more accurate than its rivals in both situations.

The proposed three-stage technique also applies to other types of measurements. A future research direction is to find out how much improvement this technique brings to measurements using Bluetooth and ultra-wideband signals. How to fuse different types of measurements, and how much additional improvement this may bring, will be of interest too. The maximum likelihood Kalman filter method that we use in the dynamic positioning stage is only valid if the motion model of the receiver is linear and Gaussian. However, non-linear motion models are sometimes used, particularly in robotics. An extension of the proposed method consists in modifying the dynamic localization stage so as to handle these kinds of non-linear models. While this is out of the scope of the present work, it is a future research direction as well.

## Figures and Tables

**Table 1:** Positioning error (empty room).

Methods	Mean Error [meters]	Minimum Error [meters]
Static positioning	1.0970	0.3196
PhaseFi	1.4722	0.5021
FILA	2.3825	0.5511
DeepFi	2.0283	0.3210

**Table 2:** Positioning error (office).

Methods	Mean Error [meters]	Minimum Error [meters]
Static positioning	1.4551	0.2763
PhaseFi	1.8722	0.4395
FILA	2.5826	0.4863
DeepFi	2.6770	1.0355

**Table 3:** Tracking Result.

Methods	Mean Error [meters]	Maximum Error [meters]
Static positioning	0.9879	2.4325
Particle filter	1.6475	2.8131
Dynamic positioning	0.4602	1.0706
